# Postmortem distribution of mepirapim and acetyl fentanyl in biological fluid and solid tissue specimens measured by the standard addition method

**DOI:** 10.1007/s11419-018-0431-z

**Published:** 2018-07-07

**Authors:** Akira Mochizuki, Hiroko Nakazawa, Noboru Adachi, Kenichi Takekawa, Hideki Shojo

**Affiliations:** 1 Forensic Science Laboratory, Yamanashi Prefectural Police Headquarters, 312-4 Kubonakajima, Isawa, Fuefuki, Yamanashi 406-0036 Japan; 2 Department of Legal Medicine, Graduate Faculty of Interdisciplinary Research, University of Yamanashi, 1110 Shimokato, Chuo, Yamanashi 409-3898 Japan

**Keywords:** Mepirapim, Synthetic cannabinoid, Acetyl fentanyl, Postmortem distribution, LC–MS, Standard addition method

## Abstract

**Purpose:**

Mepirapim is a new synthetic cannabinoid. We previously reported that the concentrations of unchanged mepirapim in whole blood and urine were much higher than those of other synthetic cannabinoids. To determine the postmortem distribution of mepirapim and acetyl fentanyl in the deceased individual, we established a standard addition method for detailed analysis by liquid chromatography–mass spectrometry (LC–MS) for quantification of these drugs.

**Methods:**

The LC–MS method was fully validated for linearity, extraction recovery, matrix effect and repeatability.

**Results:**

Good linearities, extraction recoveries, matrix effects and repeatabilities were shown for both target compounds in all specimens. The concentrations of mepirapim and acetyl fentanyl in three body fluid specimens and 12 solid tissue specimens were measured. For mepirapim, the highest concentrations were found in the liver and kidney, and the concentrations in the blood and urine specimens were one order of magnitude lower than the high concentrations in the solid tissues except the psoas major muscle. For acetyl fentanyl, the highest concentrations were found in the myocardium, spleen and kidney, and the concentrations in the body fluid specimens were also one order of magnitude lower than the highest concentrations in the solid tissues. There were concentration differences of mepirapim and acetyl fentanyl among the regions of the brain.

**Conclusions:**

The concentration of unchanged mepirapim in urine was much higher than those of other synthetic cannabinoids; the higher dosage, urinary excretion, metabolisms and/or pharmacokinetics of mepirapim may be quite different from those of other synthetic cannabinoids.

## Introduction

Illicit psychoactive substances (e.g., synthetic cannabinoids, cathinone derivatives and synthetic opioids) have become a serious threat worldwide as designer drugs of abuse [1–3]. We previously encountered a curious case in which two male subjects self-dosed mepirapim plus acetyl fentanyl by different routes of administration, that is, intravenously and by inhalation [4]. Mepirapim is a new and unique synthetic cannabinoid that was first identified in herbal blends in Japan [5]. Very recently, the affinities of mepirapim toward CB_1_ and CB_2_ in term of *K*_i_ values have been reported. The *K*_i_ values were 2650 and 1850 nM for CB_1_ and CB_2_, respectively [6], but it actually functions as a CB receptor agonist. This compound differs from JWH-018, because it has a 4-methylpiperazine group in place of the naphthyl group [4]. We thus reported a detailed gas chromatography–tandem mass spectrometry with the internal standard (IS) method for quantification of mepirapim and acetyl fentanyl in whole blood and urine [4]; the concentrations of unchanged mepirapim in whole blood and urine were much higher than those of other common synthetic cannabinoids.

On the other hand, acetyl fentanyl is a synthetic fentanyl analog in which the propionyl group of fentanyl is replaced by an acetyl group [7]. Recently, acetyl fentanyl has been encountered in clinical and forensic case studies [8–15]. The number of available reports dealing with determination of acetyl fentanyl from multiple specimens in authentic cases has been limited [3, 10, 11, 15]. In addition, the number of specimens was few; one report included five specimens [11], and another one included four specimens [15]. The most detailed report was provided by Poklis et al. [10], including seven specimens using 14 fatal cases. In this study, we have quantified both mepirapim and acetyl fentanyl in as many as 15 specimens. To our knowledge, this is the most detailed study to date for the distribution of acetyl fentanyl, and also is the first demonstration of distribution of mepirapim in an authentic fatal poisoning case. Such detailed investigation of postmortem distribution is very useful for evaluating the cause(s) of death. In addition, we have used the standard addition method for analysis by liquid chromatography–mass spectrometry (LC–MS), which can overcome the different matrix effects and recovery rates in different specimens, and also does not need blank human matrices for validation experiments.

## Case history

The deceased in the autopsy case used in our analysis was one of two subjects who had abused drugs together in December 2013. According to the confession statement of the surviving individual, the deceased individual (a male in his 60s) self-administered the drug (approximately 50–60 mg) via intravenous injection. Approximately 10 h after dosing, the surviving individual recognized that the other individual had died. An autopsy was performed, and postmortem biological fluid (heart whole blood, femoral vein whole blood and urine) and solid (cerebrum, cerebellum, pons, medulla oblongata, lung, myocardium, liver, pancreas, kidney, adrenal gland, spleen and psoas major muscle) tissue specimens were collected. The postmortem interval was estimated to be 64 h, but because the cadaver had been stored at 4 °C until autopsy, it was relatively fresh at autopsy. All specimens were stored at − 80 °C until analysis. The ‘Angela’ drug product found at the scene was seized and analyzed; the powder consisted of 73.2 ± 0.4% mepirapim and 18.9 ± 0.2% acetyl fentanyl (w/w) [4]. Drug analyses from the deceased were performed at our department by the request of judicial authorities.

## Materials and methods

### Materials

Mepirapim hydrochloride, acetyl fentanyl, JWH-200 and acetyl fentanyl-*d*_5_ were purchased from Cayman Chemical (Ann Arbor, MI, USA), and Isolute SLE + (1 mL capacity) columns from Biotage (Uppsala, Sweden). Other common chemicals used in this study were of the highest purity commercially available.

### Extraction procedure

To 50 µL of each body fluid or 100 µL solid tissue homogenate (2 g tissue in 8 mL 0.01 N HCl solution thoroughly homogenized with a blender) were added 20 µL of methanol solution containing a known amount of mepirapim and acetyl fentanyl as standard additions, 20 µL of methanol solution containing 50 ng of JWH-200 and 10 ng of acetyl fentanyl-*d*_5_ as ISs for mepirapim and acetyl fentanyl, respectively, and 0.75 mL of 1% Na_2_CO_3_. The mixture was mixed on a vortex mixer and transferred onto an Isolute SLE + (1 mL capacity) column. The column was eluted by using 5 mL of methyl *tert*-butyl ether. The eluent was evaporated, and the residue was reconstituted in 200 µL methanol. A 2-µL aliquot was used for analysis by LC–MS.

### LC–MS conditions

Quantitative analysis was performed by selected-ion monitoring (SIM) analysis of LC–MS. Briefly, LC–MS was performed on an ACQUITY Arc LC system connected to an ACQUITY QDa mass spectrometer (Waters, Milford, MA, USA). The LC conditions were as follows: XBridge BEH C18 column (75 × 3.0 mm i.d., particle size, 2.5 µm; Waters); injection volume, 2 µL; flow rate, 0.5 mL/min; elution mode, isocratic with 10 mM ammonium acetate in water/methanol (45:55, v/v); column temperature, 40 °C. The MS conditions were as follows: interface, electrospray ionization mode; polarity, positive; probe temperature, 600 °C; capillary voltage, 1.2 kV; scan mode, SIM; monitoring ion and cone voltage, *m/z* 314 and 10 V for mepirapim, *m/z* 323 and 20 V for acetyl fentanyl, *m/z* 385 and 20 V for JWH-200 and *m/z* 328 and 20 V for acetyl fentanyl-*d*_5_, respectively.

### Standard addition method

The standard addition method can completely overcome matrix effects. The method does not require blank human body fluid or solid tissue specimens that are negative for target compounds as described before. Note that when investigating the distribution of a xenobiotic compound with different types of matrices, the standard addition method is required to construct a calibration curve to obtain a single concentration value [16, 17]. To obtain each value, six different concentrations of the target compounds and fixed concentrations of ISs were added to homogenate portions of the same matrix, respectively, for construction of a calibration curve. The slope of the calibration curve crosses the *y*-intercept (intensity) and intersects the *x*-axis (target compound concentration) at the negative side; the absolute value of which indicates the preexisting concentration of the target compounds in the matrix.

### Determination of matrix effects and recovery rates

To determine the matrix effects and recovery rates for mepirapim and acetyl fentanyl in body fluid and solid tissue specimens, we used the method reported previously [16, 17]. Briefly, we first measured all concentrations of the two target compounds in matrices by using the standard addition method. Then, the same extraction procedure without standard addition was again performed twice for each matrix. At the final step of the extraction, in which 200 µL of methanol was added to reconstitute the extract residue after evaporation of the eluent, we used 200 µL of pure methanol (unspiked) in one sample and also used 200 µL of a methanol solution (spiked) containing the same amounts of mepirapim and acetyl fentanyl contained in the initial 50 µL body fluid or 100 µL solid tissue homogenate as those already measured by the standard addition method for each cadaver specimen in another sample. After reconstitution with the methanol solutions, 2 µL aliquots were injected into the LC–MS system. The peak area obtained by reconstitution with pure methanol was designated as B; the peak area obtained by reconstitution with methanol containing the same amount of the target compound in a specimen of the deceased was designated as A. A 2-µL volume of the above methanol solution (neat sample) containing the same amount as that contained at the initial step without any reconstitution was injected into the LC–MS system; the resulting peak area was designated as C.

Each matrix effect and recovery rate was calculated according to the following equations:

$${\text{Matrix effect }}\left( \% \right) = \left[ {\left( {A - B} \right)/C} \right] \times 100$$

$${\text{Recovery rate }}\left( \% \right) = \left[ {B/\left( {A - B} \right)} \right] \times 100$$

## Results

### Calibration curves

To confirm the linearity of the calibration curve for mepirapim and acetyl fentanyl, the standard addition calibration curves were constructed in suitable concentration ranges as shown in Table 1 for the body fluid and solid tissue specimens by using six plot points at different concentrations each (*n* = 5 each). Good linearity was obtained for both compounds and for all specimens, with correlation coefficients not smaller than 0.9941 (Table 1).

**Table 1:** Standard addition calibration equations for mepirapim and acetyl fentanyl in body fluid and solid tissue specimens obtained from the deceased

Specimen	Mepirapim			Acetyl fentanyl		
	Range (ng/mL or g)	Equation	Correlation coefficient (*r*^2^)	Range (ng/mL or g)	Equation	Correlation coefficient (*r*^2^)
Heart whole blood	56.7–3013	*y *= 0.000829*x *+ 0.486	0.9983	15.5–827	*y *= 0.00528*x *+ 1.11	0.9993
Femoral vein whole blood	59.3–3151	*y *= 0.000839*x *+ 0.464	0.9998	12.5–664	*y *= 0.00509*x *+ 0.865	0.9996
Urine	52.7–2801	*y *= 0.000913*x *+ 0.281	0.9991	12.6–670	*y *= 0.000553*x *+ 0.933	0.9995
Cerebrum	234–2614	*y *= 0.00183*x *+ 1.13	0.9949	60.5–675	*y *= 0.0109*x *+ 1.59	0.9992
Cerebellum	231–2467	*y *= 0.00178*x *+ 0.953	0.9941	59.6–636	*y *= 0.00962*x *+ 1.32	0.9992
Pons	271–2921	*y *= 0.00175*x *+ 1.17	0.9959	72.2–778	*y *= 0.0113*x *+ 1.88	0.9985
Medulla oblongata	169–1860	*y *= 0.00197*x *+ 0.681	0.9995	43.9–484	*y *= 0.0104*x *+ 1.03	0.9998
Lung	446–5245	*y *= 0.00154*x *+ 0.887	0.9973	36.8–432	*y *= 0.0101*x *+ 0.967	0.9997
Myocardium	417–4489	*y *= 0.00150*x *+ 0.947	0.9984	113–1216	*y *= 0.0100*x *+ 2.39	0.9990
Liver	502–5153	*y *= 0.00150*x *+ 1.86	0.9998	35.5–364	*y *= 0.0114*x *+ 0.933	0.9991
Pancreas	170–1984	*y *= 0.00178*x *+ 0.939	0.9948	82.0–959	*y *= 0.0106*x *+ 2.32	0.9998
Kidney	825–9208	*y *= 0.00131*x *+ 1.47	0.9964	93.7–1047	*y *= 0.0101*x *+ 2.38	0.9982
Adrenal gland	183–1963	*y *= 0.00234*x *+ 0.741	0.9998	49.4–530	*y *= 0.0104*x *+ 1.01	0.9999
Spleen	324–3559	*y *= 0.00151*x *+ 1.12	0.9999	100–1100	*y *= 0.0102*x *+ 2.40	0.9998
Psoas major muscle	234–2538	*y *= 0.00157*x *+ 0.249	0.9992	27.7–300	*y *= 0.0121*x *+ 0.686	0.9997

Value is the mean of five determinations

### Validation of the method

**Fig. 1:**
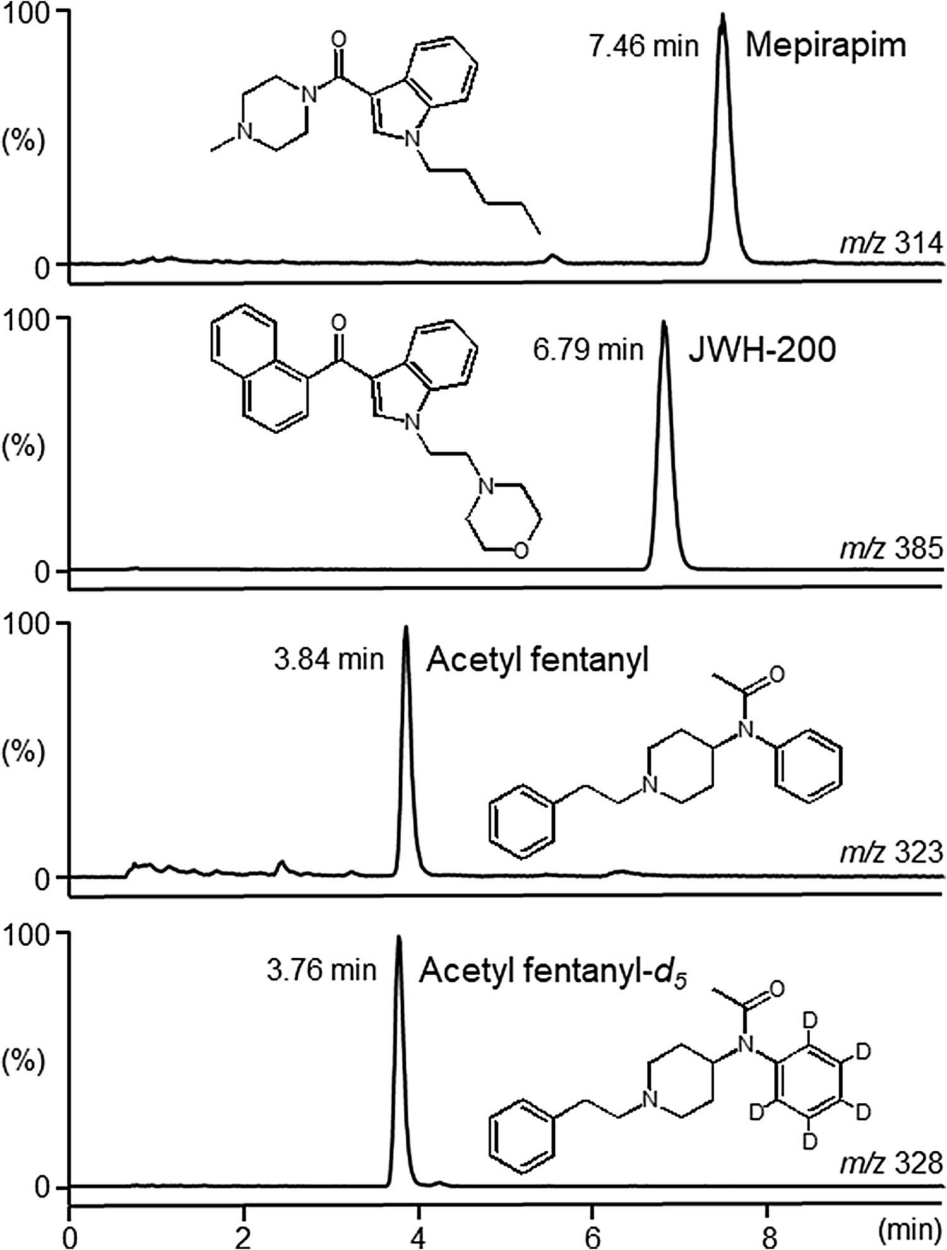
Selected-ion monitoring chromatograms for mepirapim, acetyl fentanyl and internal standards JWH-200 and acetyl fentanyl-*d*_5_ in the extract of femoral vein whole blood, recorded by liquid chromatography–mass spectrometry

Figure 1 shows the SIM chromatograms of mepirapim and acetyl fentanyl extracted from femoral vein whole blood, and JWH-200 and fentanyl-*d*_5_ spiked into the whole blood as ISs. The chromatograms showed very symmetrical peaks for the target compounds and low backgrounds in all specimens.

Given that the standard addition method was used in this study, it was impossible to present the usual accuracy and precision data using blank human specimens spiked with different concentrations of mepirapim and acetyl fentanyl (quality controls), because we did not use blank human specimens obtained from another suitable autopsy for ethical reasons. Instead, as shown in Table 2, we tested the intraday and interday repeatability for the two target compounds in femoral vein whole blood and liver specimens as examples. For mepirapim, the repeatability expressed as relative standard deviations was ≤ 10.7%. For acetyl fentanyl, the repeatability was ≤ 10.8%.

**Table 2:** Examples of intraday and interday repeatability for determination of mepirapim and acetyl fentanyl in femoral vein whole blood and liver specimens of the deceased

Specimen	Compound	Intraday (*n* = 5)		Interday (*n* = 5)	
		Concentration found (ng/mL or g)^a^	Repeatability (%RSD)	Concentration found (ng/mL or g)^a^	Repeatability (%RSD)
Femoral vein whole blood	Mepirapim	554 ± 46.0	8.31	541 ± 23.1	4.27
	Acetyl fentanyl	170 ± 9.30	5.47	173 ± 9.74	5.63
Liver	Mepirapim	6300 ± 405	6.43	6430 ± 689	10.7
	Acetyl fentanyl	416 ± 31.0	7.47	559 ± 60.4	10.8

*RSD* relative standard deviation

^a^Data are presented as the mean ± standard deviation (SD)

We used a specially devised method for calculation of matrix effects and recovery rates, as summarized in Table 3. The matrix effects on mepirapim and acetyl fentanyl for all specimens were small, with biases of ≤ 29.6 and ≤ 13.7%, respectively (*n* = 5 each). The recovery rates of mepirapim and acetyl fentanyl were ≥ 45.6 and ≥ 62.4%, respectively (*n* = 5 each).

**Table 3:** Matrix effects and recovery rates for determination of mepirapim and acetyl fentanyl in different matrices obtained from the deceased

Specimen	Mepirapim		Acetyl fentanyl	
	Matrix effect (%bias)	Recovery (%)	Matrix effect (%bias)	Recovery (%)
Heart whole blood	− 12.2 ± 1.0	88.4 ± 11.5	− 5.8 ± 0.3	89.1 ± 7.4
Femoral vein whole blood	− 16.5 ± 2.1	80.0 ± 14.1	− 3.9 ± 0.2	81.6 ± 6.7
Urine	− 6.9 ± 0.5	75.3 ± 5.4	− 4.3 ± 0.3	89.9 ± 9.1
Cerebrum	− 5.5 ± 0.5	45.6 ± 0.9	2.8 ± 0.3	73.8 ± 3.1
Cerebellum	− 5.8 ± 0.1	76.0 ± 2.0	− 2.1 ± 0.1	84.6 ± 1.6
Pons	+ 1.7 ± 0.03	69.6 ± 1.3	+ 5.9 ± 0.2	81.3 ± 1.7
Medulla oblongata	+ 8.1 ± 1.2	79.8 ± 15.9	+ 11.6 ± 1.1	81.5 ± 10.5
Lung	− 10.7 ± 0.5	76.8 ± 5.6	+ 5.7 ± 0.2	78.1 ± 3.6
Myocardium	− 11.5 ± 0.5	63.5 ± 5.1	− 2.1 ± 0.1	72.4 ± 2.1
Liver	− 19.0 ± 1.2	63.9 ± 6.7	+ 1.0 ± 0.03	87.6 ± 4.0
Pancreas	− 12.1 ± 2.0	71.1 ± 10.4	+ 13.7 ± 1.5	87.9 ± 10.7
Kidney	− 19.6 ± 1.7	66.5 ± 7.0	− 4.8 ± 0.4	87.5 ± 13.4
Adrenal gland	− 29.6 ± 2.8	67.1 ± 8.0	+ 5.2 ± 0.4	62.4 ± 3.0
Spleen	− 9.5 ± 1.1	75.3 ± 7.1	− 4.8 ± 0.4	92.2 ± 7.0
Psoas major muscle	− 12.2 ± 1.3	58.9 ± 6.9	− 2.1 ± 0.1	84.4 ± 4.2

Data are presented as the mean ± SD (*n *= 5 each). The detailed method for obtaining the values of matrix effects and recovery rates is given in the text

By dilution of each specimen, the detection limits of mepirapim and acetyl fentanyl by this method were estimated to be approximately 20 and 2 ng/mL or g, respectively.

### Postmortem concentrations of mepirapim and acetyl fentanyl in body fluid and solid tissue specimens

As shown in Table 4, the concentrations of mepirapim in heart whole blood, femoral vein whole blood and urine were as high as 587, 554 and 309 ng/mL, respectively, as compared with those of other common synthetic cannabinoids. The concentrations in the solid tissue specimens except psoas major muscle were one order of magnitude higher than those of the blood and urine specimens. The highest concentrations of mepirapim among these specimens were found in the liver and kidney at 5410–6300 ng/g.

**Table 4:** Postmortem concentrations of mepirapim and acetyl fentanyl in body fluid and solid tissue specimens obtained from the deceased

Specimen	Mepirapim (ng/mL or g)	Acetyl fentanyl (ng/mL or g)
Heart whole blood	587 ± 42	212 ± 15
Femoral vein whole blood	554 ± 46	170 ± 9
Urine	309 ± 44	169 ± 15
Cerebrum	2740 ± 185	649 ± 34
Cerebellum	2690 ± 316	688 ± 28
Pons	3300 ± 162	821 ± 5
Medulla oblongata	1710 ± 41	489 ± 22
Lung	2720 ± 339	448 ± 28
Myocardium	3120 ± 332	1180 ± 82
Liver	6300 ± 405	416 ± 31
Pancreas	2400 ± 325	987 ± 43
Kidney	5410 ± 574	1140 ± 140
Adrenal gland	1580 ± 194	481 ± 26
Spleen	3610 ± 287	1150 ± 32
Psoas major muscle	792 ± 122	281 ± 11

Data are presented as the mean ± SD (*n* = 5 each)

For acetyl fentanyl, the concentrations in heart whole blood, femoral vein whole blood and urine were 212, 170 and 169 ng/mL, respectively. The highest concentrations of acetyl fentanyl among these specimens were found in the myocardium, followed by the spleen and kidney at 1140–1180 ng/g.

Within the brain, both mepirapim and acetyl fentanyl distributed unevenly. The highest concentrations of both compounds were found in the pons, followed by the cerebrum or cerebellum. The lowest concentrations were found in the medulla oblongata for both compounds.

## Discussion

In this study, we quantified mepirapim and acetyl fentanyl in body fluid and solid tissue specimens taken from the deceased individual by LC–MS using the standard addition method. The concentrations of mepirapim (554–587 ng/mL) and acetyl fentanyl (170–212 ng/mL) in blood samples were similar to those of our previous study using the usual IS calibration method (mepirapim, 563–593 ng/mL and acetyl fentanyl, 125–155 ng/mL) [4]. As described in our previous report, the concentrations of mepirapim in blood specimens were much higher than those of various other synthetic cannabinoids (0.1–199 ng/mL) in previous reports of poisoning deaths [2, 18–20]. On the other hand, the concentrations of acetyl fentanyl in the femoral vein and heart whole blood were at levels relatively similar to those of previous reports; the acetyl fentanyl blood levels in fatal cases were 6–600 ng/mL [10], 250–260 ng/mL [11], 153 ng/mL [12], 270 ng/mL [13], 192–285 ng/mL [14] and 235 ng/mL [15].

The urinary level of unchanged mepirapim in the deceased individual was also as high as 309 ng/mL, although the concentration was slightly lower than that previously found (527 ng/mL) [4]. In addition, our previous study showed that the urine concentration of unchanged mepirapim in a surviving individual was much higher than expected (4900 ng/mL) [4]. Such a high concentration has never been observed for other synthetic cannabinoids; the levels of unchanged synthetic cannabinoids in human urine specimens are generally very low at subnanograms/mL [21]. Further, the concentrations of unchanged mepirapim in the postmortem solid tissue specimens (792–6300 ng/g) were also much higher than expected. Among these tissue samples, the highest levels of unchanged mepirapim were found in the liver (6300 ng/g) and kidney (5410 ng/g), where drugs are usually metabolized and excreted into urine. It is unknown whether the vendors recommended the users to take high doses of mepirapim to gain sufficient drug effects; actually the affinities of mepirapim for both CB_1_ and CB_2_ receptors were low [6]. The most important problem for mepirapim is the toxicity of the compound; toxicity, pharmacokinetics and metabolism of this compound are all unknown and remain to be explored.

The urinary level of acetyl fentanyl was close to those in femoral vein and heart whole blood, which suggested that death occurred before sufficient excretion and metabolism of the drug. Moreover, the concentrations in urine were lower than those in the liver and brain, which suggested that death was rapid [10, 11]. Further, our detailed analyses revealed that the highest levels of acetyl fentanyl were distributed to the myocardium, kidney and spleen rather than to the liver and brain. These postmortem distribution results may provide new insight into the excretion, pharmacokinetics and metabolism of acetyl fentanyl in a case of fatal poisoning after intravenous exposure, although previous reports dealing with the distribution of acetyl fentanyl did not include the myocardium and kidney [10, 11, 15].

## Conclusions

To quantify the postmortem distribution of mepirapim and acetyl fentanyl in a drug user, we established a detailed procedure for LC–MS analysis with the standard addition method. To our knowledge, this is the first study on the postmortem distribution of mepirapim. The concentrations of unchanged mepirapim in the postmortem specimens were quite different from those of other synthetic cannabinoids and much higher than expected. Further, the highest levels of mepirapim were distributed to the liver and kidney, whereas those of acetyl fentanyl were distributed to the myocardium, kidney and spleen rather than to the liver and brain. Also for acetyl fentanyl, this is the most detailed distribution study so far reported, to our knowledge. The detailed studies on distribution of drugs or poisons give various information and/or confirmation of the cause of death, antemortem interval, rough estimation of administered dose and route of administration.
